# Cytotoxicity activities and chemical characteristics of exopolysaccharides and intracellular polysaccharides of *Physarum polycephalum* microplasmodia

**DOI:** 10.1186/s12896-021-00688-5

**Published:** 2021-03-27

**Authors:** Tuyen T. H. Do, Tran N. B. Lai, Steven L. Stephenson, Hanh T. M. Tran

**Affiliations:** 1 School of Biotechnology, International University, Ho Chi Minh City, Vietnam; 2 Vietnam National University, Ho Chi Minh City, Vietnam; 3 Faculty of Biotechnology, Ho Chi Minh City University of Food Industry, Ho Chi Minh City, Vietnam; 4 Department of Biological Sciences, University of Arkansas, Fayetteville, AR USA

**Keywords:** *Physarum polycephalum*, Polysaccharides, Cytotoxicity, MCF-7, HeLa, Microplasmodia

## Abstract

**Background:**

Microbial polysaccharides have been reported to possess remarkable bioactivities. *Physarum polycephalum* is a species of slime mold for which the microplasmodia are capable of rapid growth and can produce a significant amount of cell wall-less biomass. There has been a limited understanding of the polysaccharides produced by microplasmodia of slime molds, including *P. polycephalum*. Thus, the primary objectives of this research were first to chemically characterize the exopolysaccharides (EPS) and intracellular polysaccharides (IPS) of *P. polycephalum* microplasmodia and then to evaluate their cytotoxicity against several cancer cell lines.

**Results:**

The yields of the crude EPS (4.43 ± 0.44 g/l) and partially purified (deproteinated) EPS (2.95 **±** 0.85 g/l) were comparable (*p* > 0.05) with the respective crude IPS (3.46 ± 0.36 g/l) and partially purified IPS (2.45 ± 0.36 g/l). The average molecular weight of the EPS and IPS were 14,762 kDa and 1788 kDa. The major monomer of the EPS was galactose (80.22%), while that of the IPS was glucose (84.46%). Both crude and purified IPS samples showed significantly higher cytotoxicity toward Hela cells, especially the purified sample and none of the IPSs inhibited normal cells. Only 38.42 ± 2.84% Hela cells remained viable when treated with the partially purified IPS (1 mg/ml). However, although only 34.76 ± 6.58% MCF-7 cells were viable when exposed to the crude IPS, but the partially purified IPS displayed non-toxicity to MCF-7 cells. This suggested that the cytotoxicity toward MCF-7 would come from some component associated with the crude IPS sample (e.g. proteins, peptides or ion metals) and the purification process would have either completely removed or reduced amount of that component. Cell cycle analysis by flow cytometry suggested that the mechanism of the toxicity of the crude IPS toward MCF-7 and the partially purified IPS toward Hela cells was due to apoptosis.

**Conclusions:**

The EPS and IPS of *P. polycephalum* microplasmodia had different chemical properties including carbohydrate, protein and total sulfate group contents, monosaccharide composition and molecular weights, which led to different cytotoxicity activities. The crude and partially purified IPSs would be potential materials for further study relating to cancer treatment.

**Supplementary Information:**

The online version contains supplementary material available at 10.1186/s12896-021-00688-5.

## Background

Polysaccharides are biopolymers comprised of monosaccharides [1] that are essential constituents of all living organisms and are associated with a variety of vital functions necessary to sustain life [2]. Polysaccharides can be classified into three different main groups according to their locations. These are (a) cytosolic or intracellular polysaccharides (IPSs) which are inside cells and serve as carbon and energy sources for the cells; (b) structural polysaccharides that make up the cell walls, including peptidoglycans, techoid acids, and lipopolysaccharides; and (c) exopolysaccharides (EPSs) that are excreted to the extracellular environment in the form of capsules or slime [2].

In general, polysaccharides possess various physicochemical properties, including gelation [1] as well as film-forming capability, viscosity and stability [3]. These properties depend upon their composition and molecular architecture [4]. Recently, accumulated evidence has demonstrated that polysaccharides have a broad spectrum of biological effects, such as antibiotic, antioxidant, anti-mutant, anticoagulant, and immune stimulation activities [5–7]. The bioactivity of a given polysaccharide is closely related to its structure and physicochemical properties [8]. In terms of anticancer properties, the antitumor effects of polysaccharides have been reported to depend upon the molecular weight, chemical composition, structure of the polymeric backbone, and degree of branching [9, 10]. Recent studies have suggested that polysaccharides that possess high anticancer activities share some common physical-chemical properties, including having a relatively high molecular weight, expanded chains, a specific conformation, and a complicated monosaccharide composition. The latter characteristics cause them to have more opportunities to collide and bind with the receptors on the surface of tumor cells [11, 12]. Numerous studies on polysaccharides from microorganisms—including bacteria, fungi, and microalgae—have been carried out [1, 11, 13].

Slime molds (myxomycetes) are a group of unique protozoans for which the common name (i.e., “slime molds”) most often used is based on the fact that they excrete noticeable amounts of slimy materials in both liquid and solid culture [14]. Among the myxomycetes, those members of the order Physarales (e.g., *P. polycephalum*) often form large plasmodia and are relatively easy to culture on synthetic media. When cultured in liquid media, microplasmodia are formed instead of plasmodia [14]. Plasmodia and microplasmodia possess several important characteristics, including having no cell walls, rapid growth, and probably sharing the same set of metabolites. However, the remarkable advantage of microplasmodia is that as they can grow in a liquid medium, thus making large-scale culture easier.

Since slime molds are unusual organisms as a result of having characteristics of both fungi and protozoans, they have the source of a large number of novel bioactive compounds. As such, it would be anticipated that they would also produce some unique polysaccharides. In fact, there has been only limited information available on polysaccharides from both plasmodia and microplasmodia of *P. polycephalum* except for a few reports in the 70s and early 90s. For example, Simon and Henney [15] and McCormick et al. [16] found that exopolysaccharides produced by microplasmodia of *P. polycephalum* are glycoproteins composed of galactose, sulfate, and trace amounts of rhamnose. In another study, the EPSs were found to consist of two galactans with different ratios of phosphorus and sulfur content [17]. In terms of biological activities, initial data demonstrated that the EPSs could inhibit cell growth and division of *Bacillus subtilis* [18]*.*

A more recent study carried out with plasmodia of *P. polycephalum* and *Ph. oblonga* found that the crude exopolysaccharides of the plasmodia showed significant antifungal activities against *Candida albicans* and cytotoxicity activities toward HepG2 (liver carcinoma cell line) [14].

In general, there have not been any reports on the cytotoxicity activities of both intracellular polysaccharides (IPSs) and exopolysaccharides (EPSs) from *P. polycephalum* microplasmodia. Therefore, the primary objectives of the present study were first to chemically characterize the IPS and EPS from *P. polycephalum* and then to investigate their cytotoxicity activities against different cancer cell lines. Since this was a preliminary study, both crude and partially purified samples were included for comparison.

## Results

### Yields of different types of polysaccharides from *P. polycephalum* microplasmodial culture

The amounts of crude and partially purified EPSs and IPSs obtained from 5-day-old microplasmodial cultures of *P. polycephalum* are shown in Table 1.

**Table 1:** Yields of different types of polysaccharide from *P. polycephalum*

Parameter	Amount (g/l)
Dried microplasmodial biomass*	**11.67 ± 0.52**
Crude EPS	4.43^a^ ± 0.44
Partially purified EPS	2.95^a^ **±** 0.85
Crude IPS	**3.46^a^ ± 0.36 (0.30 g/g dried biomass)
Partially purified IPS	**2.45^a^ ± 0.36 (0.21 g/g dried biomass)

**indicates the amount of the intracellular polysaccharides obtained from one liter of the culture medium (g/l). These figures were calculated based on their contents in one gram of the dried biomass (data inside the brackets), and the total dried biomass obtained from one liter of the culture medium*. The same letter (a) indicates insignificant difference (*p* > 0.05)

The TCA treatment and the dialysis process seemed to successfully remove the unbound proteins and some impurities. As a result, the amount of the partially purified EPS was lower than that of the crude EPS and the same approach was applied to the IPS samples. Remarkably, the amounts of EPSs and IPSs were comparable either in crude or partially purified forms (*p* > 0.05).

### Chemical composition and functional group analysis of the polysaccharides

The total carbohydrate contents of EPSs and IPSs were determined by the phenol-sulfuric acid method [19]. Protein and sulfate group content of each polysaccharide sample was analyzed by the Bradford assay [20] and the barium-chloride gelatin method [21] as previously described.

In general, the carbohydrate content of the crude IPSs was slightly higher than that of the EPSs samples (*p* > 0.05). However, the purified IPS had a significantly higher content of carbohydrate (*p* < 0.05) compared to those of the EPSs samples. In addition, the contents of protein in the crude samples were similar (*p* > 0.05), but those of the purified samples were significantly lower (*p* < 0.05). This suggested that the treatment with TCA and dialysis significantly reduced the protein content of the polysaccharide samples (*p* < 0.05) (Table 2). The total carbohydrate and protein contents of all samples varied from 49.56 to 80.97%. Moreover, sulfate group content was measured only in crude samples (0.14 to 2.04%). In the partially purified samples, the sulfate groups would have been either completely removed or the amount was too small to be detected by the barium-chloride gelatin method (Table 2).

**Table 2:** Percentages of major chemical components of the polysaccharide samples from *P. polycephalum* microplasmodia

Sample	Total carbohydrate content (%)	Total protein content (%)	Total sulfate group content (%)	Total percentages
Crude EPS	44.26^a^ ± 0.70	3.26^c^ ± 0.33	2.04 ± 0.02	49.56
Partially purified EPS	56.61^a^ ± 5.73	1.16^b^ ± 0.20	ND	57.77
Crude IPS	63.38^ab^ ± 0.60	2.71^c^ ± 0.39	0.14 ± 0.02	66.23
Partially purified IPS	80.40^b^ ± 14.61	0.57^a^ ± 0.27	ND	80.97

*ND* not detected; Different letters (a, b, c, d) imply significant differences (*p* < 0.05); Total percentages: were calculated by adding together the contents of carbohydrate, protein, and sulfate (if any) detected in each sample

### FTIR-ATR (Fourier transform infrared spectroscopy – attenuated total reflectance) analysis of the polysaccharides

**Fig. 1:**
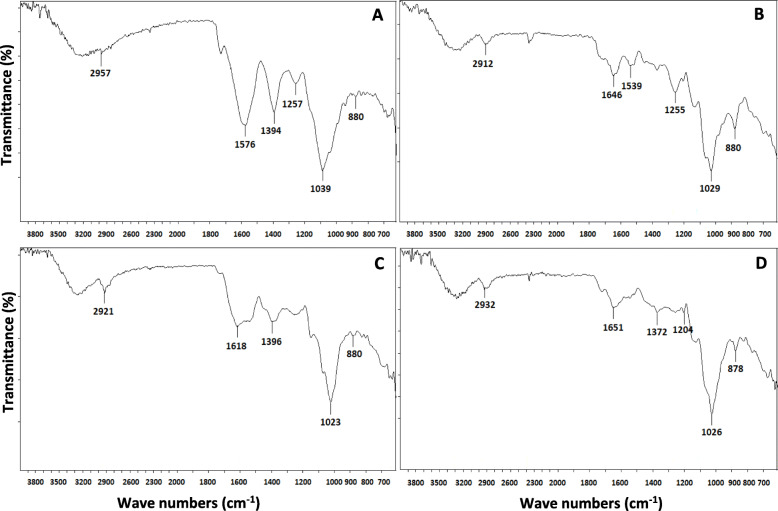
FTIR spectral analysis of the crude EPS (**a**), partially purified EPS (**b**), crude IPS (**c**); partially purified IPS (**d**)

FTIR analyses were carried out to determine the main functional groups present in the polysaccharide samples. The FTIR spectra of the four samples (Fig. 1) shared rather similar peaks in the region of 1200–1000 cm^− 1^, which were typical for the vibrations of C-O-H and C-O-C glycosidic bonds [22, 23]**.** The bands in the region of 2912–2975 cm^− 1^ were assigned to C-H stretching [24] and C-H bending vibrations [22], respectively, in polysaccharide molecules. Moreover, the peaks representing the β-glucosidic linkages in polysaccharides [25] were more clearly apparent at 880 cm^− 1^ in the partially purified EPS and IPS samples, for which the total carbohydrate contents had been found to be higher than those of the crude samples as displayed in Table 2**.** On the other hand, the less intensities of the bands, corresponding to amide I (~ 1650 cm^− 1^) and to amide II (~ 1539 cm^− 1^) vibrations [26] in the purified samples, also confirmed that the total protein contents decreased after the partial purification process.

Overall, the FTIR spectrum of the partially purified IPS was rather similar to that of *β*-D-glucan from the fruiting bodies of *Daedalea quercina* [27].

### Monosaccharide composition analysis of the polysaccharides

**Fig. 2:**
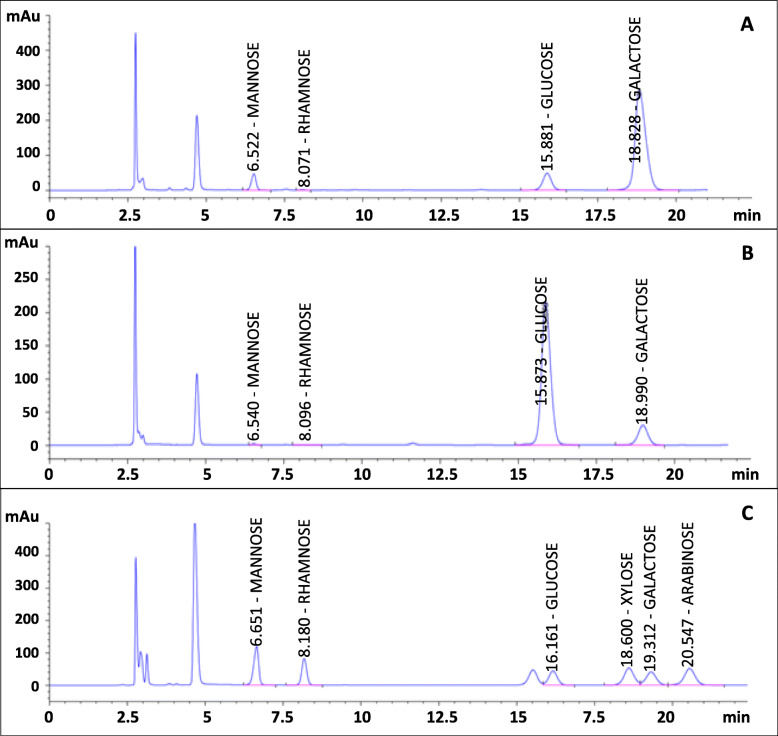
The PMP-RP-HPLC-UV chromatograms of monosaccharide analysis on the partially purified EPS (**a**), IPS (**b**), and sugar standard mixture (**c**)

Monomer composition of the partially purified EPS and IPS was analyzed by RP-HPLC-UV using a 1-phenyl-3-methyl-5-pyrazolone (PMP) pre-column derivatization procedure [28] and results are presented in Table 3 and Fig. 2. Two polysaccharides consist of glucose, galactose, mannose, and rhamnose in their overall composition but with different percentages. The main monomer of the EPS was galactose (80.22%), whereas that of the IPS was glucose (86.46%).

**Table 3:** Monosaccharide composition of the partially purified polysaccharide samples from *P. polycephalum* microplasmodia

Sample	Monosaccharide composition (%)			
	Glucose	Galactose	Mannose	Rhamnose
Partially purified EPS	13.84 ± 0.16	**80.22** ± 1.80	5.70 ± 0.15	0.24 ± 0.012
Partially purified IPS	**86.46** ± 1.50	13.22 ± 0.20	0.23 ± 0.0071	0.09 ± 0.0045

### GPC analysis of the polysaccharides

Molecular weight distribution (MWD) and average molecular weights (M_W_) of the partially purified EPS and IPS samples were determined by gel permeation chromatography (GPC) using an Agilent’s GPC/SEC software (addon Rev.B.01.01).

**Fig. 3:**
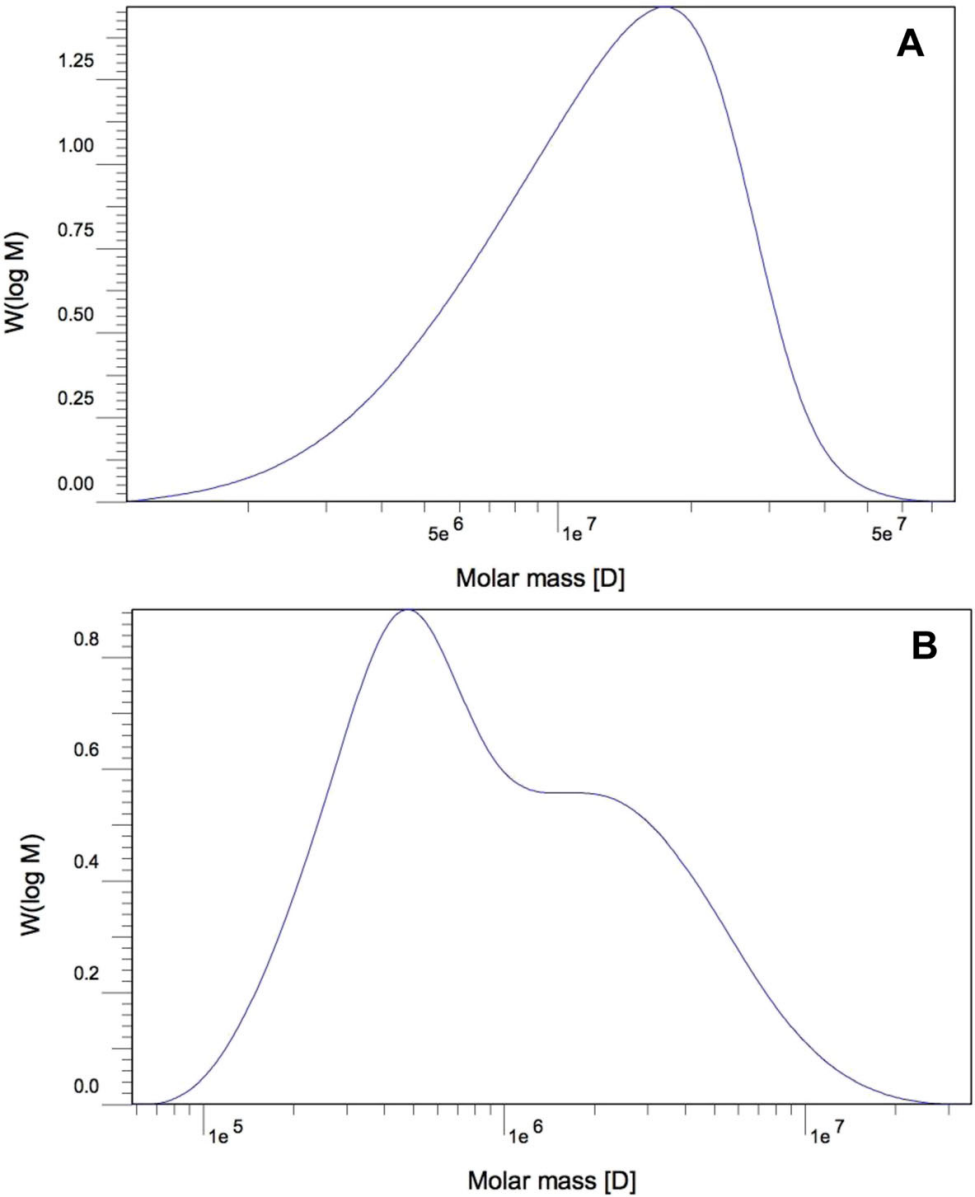
The molecular weight distributions of polysaccharide molecules in the partially purified EPS (**a**) and IPS (**b**)

The partially purified EPS showed a single distribution (Fig. 3a) with a M_W_ value of 14,762 kDa (Table 4)**.** However, the partially purified IPS displayed two different distributions (Fig. 3b) with a M_W_ value of 1788 kDa (Table 4**).**

**Table 4:** Molecular Weight (Mw), Number-Average Molecular Weight (Mn) and Polydispersity Index (PI) of *P. polycephalum* microplasmodial polysaccharides

Sample	Number weighted molecular weight (M_**n**_)	Mass-weighted molecular weight (M_**w**_)	Polydispersity (M_**n**_/M_**w**_)
Partially purified EPS	9570 kDa	14,762 kDa	1.542
Partially purified IPS	565 kDa	1788 kDa	3.160

### Cytotoxicity activities of crude and partially purified EPS and IPS samples on cancer and non-transformed cell lines

**Fig. 4:**
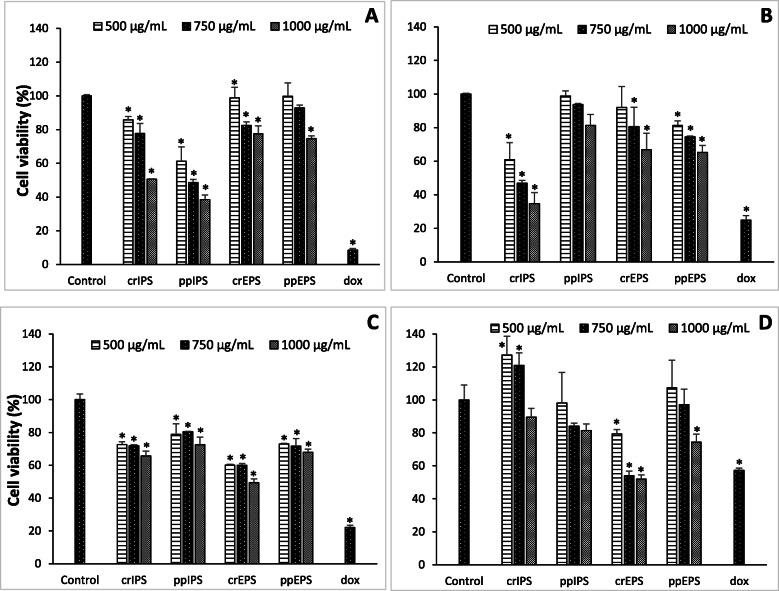
Percentages of viable HeLa (**a**), MCF-7 (**b**), HepG2 (**c**) and BAE cells (**d**) treated with 1 mg/ml polysaccharide samples. Doxorubicin (dox) 50 μg/ml was used as a positive control. Data represent the mean and standard deviation. Note that ‘cr-‘= crude sample and ‘pp-‘= partially purified sample.* indicates a significant difference as compared with the control (*p* < 0.05)

The anti-proliferation capabilities of the polysaccharide samples were tested on three cancer cell lines (MCF-7, HeLa and HepG2) and a non-transformed cell line (BAEC) using MTT assay. Sterile phosphate buffered saline was used as the negative and doxorubicin (a broad-spectrum antitumor antibiotic) with a concentration of 50 μg/ml was used as the positive control. The data obtained are shown in Fig. 4.

Doxorubicin, a broad-spectrum antitumor antibiotic, has been one of the most extensively used agents in the chemotherapy regimens of cancer patients [29]. In the present study, doxorubicin 50 μg/ml showed anti-proliferative activities toward all the tested cell lines, with the highest rate of 91.55% (towards HeLa) and lowest rate of 42.75% (towards BAEC). This anti-cancer drug expressed inhibition effects against HepG2 and MCF-7 with seemingly similar rates of 77.83 and 75.14% (*p* > 0.05). A recent study that used doxorubicin as a positive control also illustrated that HeLa is more sensitive than HepG2 to this chemotherapy drug [30].

The crude EPS was the only sample that showed inhibitory effects on non-transformed cells (BAEC) at all tested concentrations (from 0.5 to 1.0 mg/ml). Moreover, partially purified EPS at a concentration of 1.0 mg/ml decreased the viable rate of BAEC to 74.44%. No significant inhibition toward BAEC was recorded with the other samples (crude and partially purified IPS) or at lower concentrations of partially purified EPS (0.5 and 0.75 mg/ml) (Fig. 4d).

Several of the samples tested showed exceptional toxicities toward the cancer cells and the activities were dose dependent. As shown in Fig. 4a, both the crude and the partially purified IPS samples exhibited significantly high anti-proliferative activities against HeLa, but the latter sample showed higher activity than the former (Fig. 4a). At the concentration of 1.0 mg/ml, the partially purified IPS reduced the viability of HeLa to 38.42% whereas that of the crude sample was 50.56%. These values were not significantly different (*p* > 0.05). However, a significant difference in inhibitory activities against HeLa cells of these samples was recorded at the concentrations of 0.5 and 0.75 mg/ml (*p* < 0.05). On the other hand, the crude IPS displayed remarkable cytotoxicity against MCF-7. Only 34.76% of the cell population treated with 1.0 mg/ml crude IPS remained viable (Fig. 4b). This level of cell inhibition was comparable (*p* > 0.05) to that of the positive control at 50 μg/ml, since at this concentration doxorubicin reduced the viability of MCF-7 cells to 24.86%. However, it should be noted here that the partially purified IPS displayed no activity against MCF-7 except at the concentration of 1.0 mg/ml.

The data on cytotoxicity toward HepG2 revealed that crude EPS seemed to be the most effective agent, reducing the viability of cancer cells to 49.39% (Fig. 4c). However, the crude EPS also displayed almost the same level of cytotoxicity toward the normal cell line (BAEC). The other three polysaccharides expressed mild activities towards HepG2, although they showed significant toxicities towards other cancer cells, as previously mentioned.

In general, data from the MTT assay suggested that the partially purified IPS might be an effective anti-HeLa agent and the crude IPS could have the potential for MCF-7 inhibition. As such, they were evaluated in the next experiment.

### Study on the effects of IPSs on the cell cycles of cancer cells

In this experiment, HeLa cells were treated with partially purified IPS, and MCF-7 cells were treated with crude IPS. The treated cells were then stained with PI fluorochrome dye, which was followed by DNA content or cell cycle analysis by flow cytometry to study the effects of the samples on the cell cycle pattern.

One of the characteristics of apoptotic cells is the loss of nuclear DNA content due to DNA fragmentation. This results in a population of cells with reduced DNA content. PI fluorochrome dye is capable of stoichiometrically binding to DNA and the binding resulted in fluorescence emission, which is proportional to the DNA content. When apoptotic cells are stained with PI and analyzed with a flow cytometer, they will form a large peak, which can be easily discriminated from the narrow peak of cells with normal (diploid) DNA content in the red fluorescence channels [31].

**Fig. 5:**
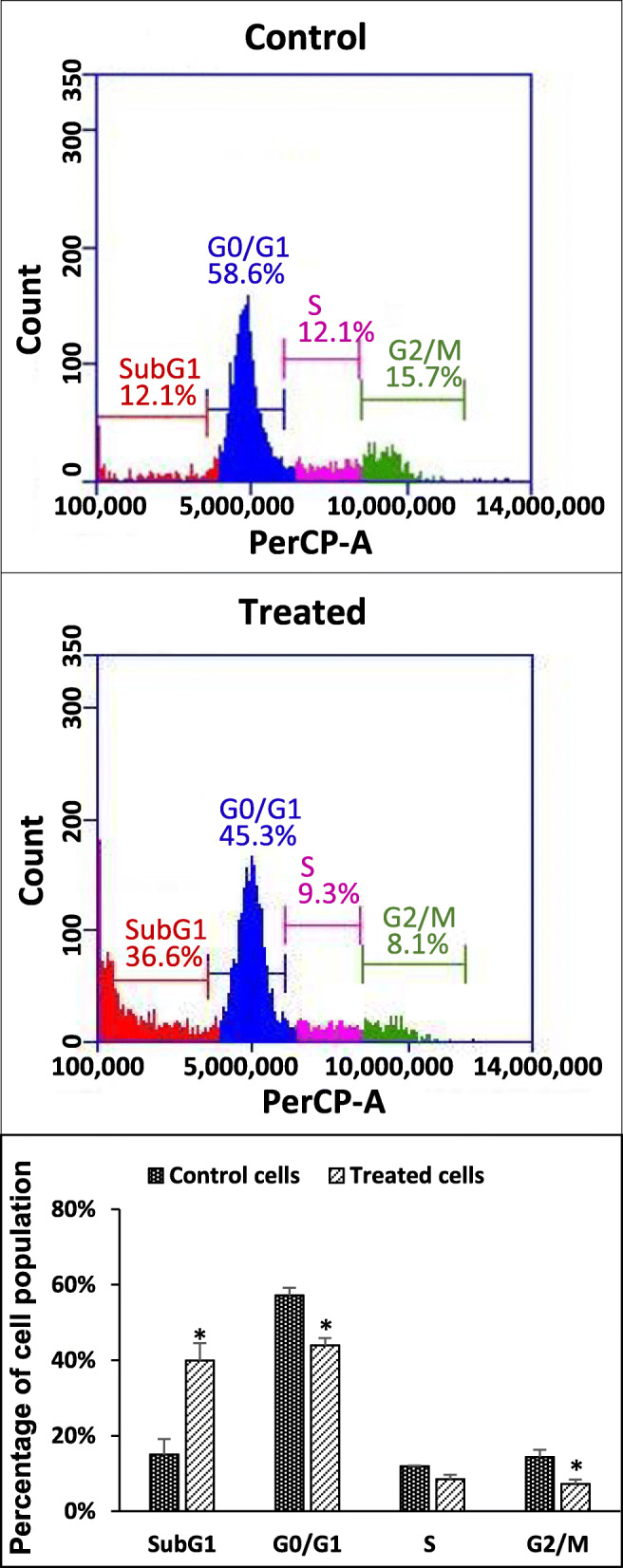
Distribution of cells in the subG1, Go/G1, S and G2/M phases of HeLa cells treated with partially purified IPS 1 mg/ml. *indicates a significant difference as compared with the control (*p* < 0.05). PBS solution was used as negative control. Data represent the mean and standard deviation

**Fig. 6:**
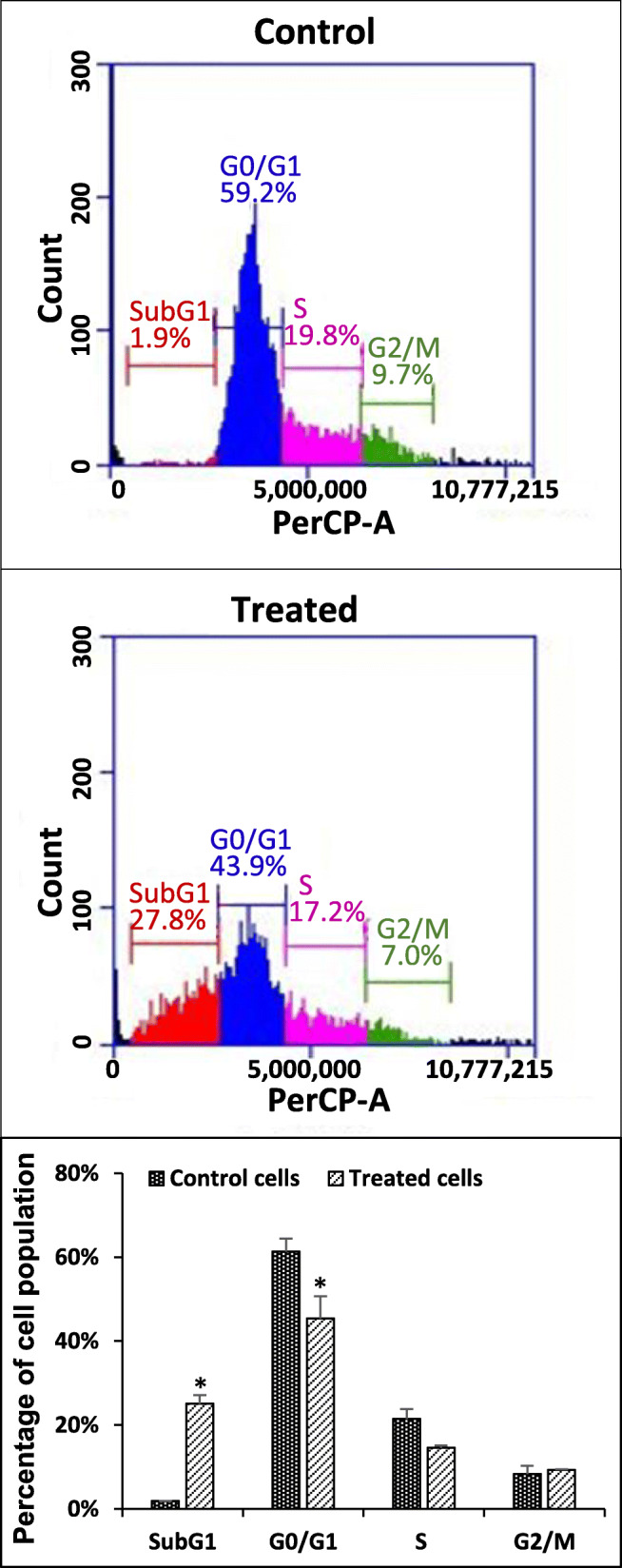
Distribution of cells in the subG1, Go/G1, S and G2/M phases of

The cell cycle patterns of the treated cancer cells, including HeLa and MCF-7, are presented in Fig. 5 and Fig. 6, respectively**.**

### MCF-7 cells treated with crude IPS 1 mg/ml

*indicates a significant difference as compared with the control (*p* < 0.05). PBS solution was used as negative control. Data represent the mean and standard deviation.

In two treatments, the cell populations of HeLa and MCF-7 at the sub-G1 stage displayed significantly higher percentages compared to that of the control (*P* < 0.05) (Figs. 5, and 6 and Supplementary data S1). Moreover, lower percentages of cells in the G1/G0, S and G2/M phases in the treatments compared to those of the controls also were recorded.

## Discussion

The amounts of EPSs and IPSs obtained from *P. polycephalum* plasmodia were comparable either in crude or partially purified forms. This would be because of the high biomass production (11.67 g/l) and the absence of cell walls in the microplasmodia, which allowed an effective extraction of the IPS. Normally, microbial IPSs are considered less advantageous compared with to EPSs because of the challenges in extracting them from the cells and also their production strongly depends on biomass production, which is normally low for most microorganisms, especially bacteria. However, it seems that with *P. polycephalum*, none of these challenges were significant. Sperl [17] reported that an amount of 5.36 g/l of EPS (7.9 g per 1.5 l of liquid medium) was obtained from a *7*-day old culture of *P. polycephalum* microplasmodia. These data are higher than what was found in the present study. However, since the purpose was to collect both the EPS and IPS, and a 5-day old culture was chosen since at day 6 to 8, little or no IPS was collected from *P. polycephalum* biomass as the microplasmodia were mostly lysed (unpublished data).

In chemical analysis assays, the closer the total percentages of three parameters (carbohydrate, protein, and sulfate contents) in a sample approached 100%, the fewer impurities or unknown components were present in the sample. Therefore, it could be concluded that the purification process had successfully eliminated the proteins and impurities from the samples as all the purified samples had the higher total percentages as compared to those of the corresponding crude samples. Huynh et al. [14] reported that the crude EPS of *P. polycephalum* plasmodia had carbohydrate and protein contents of 56.42 and 30.94%, respectively. Compared to these values, the crude EPS of the microplasmodia possessed a similar carbohydrate content but a lower protein content. This could be the nature of the plasmodia and microplasmodia or the result of differences in the physical form of the media used. Generally, the organisms could be expected to access nutrients in the liquid culture more easily than in plate cultures due to the presence of agar and lesser amount of available water.

A study of partially purified EPSs from different cyanobacteria indicated that the total sugar and protein contents of these EPSs were in the ranges of 56–87% and 1.83–4.01%, respectively [32]. The carbohydrate content of the partially purified polysaccharides from *P. polycephalum* was similar to that of the cyanobacteria. However, the protein contents of the partially purified EPS and IPS were slightly lower. In the study of crude EPS from mycelial culture of the fungus *Cordyceps sinensis*, the data obtained showed that the percentage carbohydrate varied from 54.9 to 70% and the protein from 26.2 to 29.3% [33]. Hence, in general, the amount of protein in crude EPSs of different microbes could be different, but the carbohydrate content appears similar. The differences in nutrition and energy demands among microbes could be the reason for the differences in the protein content.

It is not clear that the sulfate content detected in the crude samples was bound to the polysaccharide structures or it was just the magnesium sulfate of the culture medium. However, since the dialysis process could exclude them easily, this suggested the latter possibility. A previous study reported that crude EPSs of *P. polycephalum* and *Physarella oblonga* plasmodia (on solid media) without a dialysis treatment had higher sulfate group content (2.44 and 11.26%, respectively) [14].

The FTIR results suggested that the IPS sample may contain β-D-glucan since the IR spectrum of the IPS sample showed peaks similar to those of β-D-glucan molecules reported in the previous studies [27, 34], especially in the range of 1651–1026 cm^− 1^. This similarity, in fact, was consistent with the result of a monosaccharide analysis, which pointed out that glucose was the main component (86.46%) of the IPS, whereas for EPS it was galactose (80.22%). However, the EPS might be not related to galactan chains because when compared with the IR spectrum of potato pectic galactan, no similar characteristic was observed [35]. In addition, based on the data shown in Table 2, it would be anticipated that the presence of a large amount of unknown components in the samples might have caused some interference to the FTIR recorded spectra of the EPS samples. In terms of monosaccharide composition, there has not been any available information on the IPS composition of *P. polycephalum* microplamodia; however, in some previous studies carried out by McCormick et al. [16] and Simon and Henney [15], it was reported that *P. polycephalum* microplasmodial EPS was also mainly composed of galactose (62–72%) with a trace amount of rhamnose. The results of the present agree with these data.

Differences in anti-proliferative activities were observed among the polysaccharides from different sources (EPS and IPS) and different forms (crude and partially purified). In addition, different samples displayed different levels of cytotoxicity toward different cell lines. Specifically, the crude and especially the partially purified IPSs showed remarkable antiproliferative activities against HeLa. However, the crude IPS expressed significantly high cytotoxicity toward MCF-7, but the activity was not recorded with the partially purified IPS. The lower activity of the purified IPS sample could be explained by the possibility that the toxicity was probably due to some unbound component (e.g., unbound proteins, peptides or metal ions), and the purification process had removed or reduced this component from the crude IPS sample. In addition, the presence of sulfates in the crude IPS and distinctively higher content of carbohydrate in the partially purified IPS would contribute to the diverse nature of anti-proliferative capacities (Table 2). It is difficult to confirm if sulfate composition is the major factor that leads to the higher anti-proliferative of crude IPS as opposed to partially purified IPS since this hypothesis is not true in the case of HeLa and even for HepG2 (no significant difference, since *P* > 0.05). Moreover, at this stage of the study, there was no certain proof to declare the difference in carbohydrate amounts of the two samples had enhanced or reduced the observed activities.

Noticeably, both partially purified and crude IPSs showed no negative effects toward the non-transformed cell line (BAEC) (Fig. 4d). Generally, the substances that show effective inhibition towards cancer cells but little cytotoxicity against non-transformed cells would be considered as potential antitumor agents.

On the other hand, the anticancer activities of EPS samples were weaker than IPS samples in most cases for both crude and partially purified forms. As mentioned earlier, monomer composition could be the factor that contributes to this difference since IPS consists mainly of glucose (86.46%) and EPS consists mainly of galactose (80.22%) in their respective compositions (Table 3). The high content of glucose has been previously mentioned as a factor that had been found to contribute to high antitumor activities in several studies [25, 36]. The other possible explanation for the difference in cytotoxicity activities of the IPS and EPS samples was the existence of the β-D-glucan structure in the IPS (Fig. 1), and β-D-glucan has been detected in a number of fungal polysaccharides with significant antitumor activities [13]. Moreover, the various MW values may cause these dissimilar results since there were a number of anticancer polysaccharides with MW values around 2000 kDa [37–39], whereas there are not many records of antitumor polysaccharide with a MW value exceeding 10,000 kDa [13]. Higher molecular weight polysaccharides have been reported to exert stronger anti-tumor activities than low molecular weight fractions within certain ranges [11, 25]. This would be explained by the possibility that high MW polysaccharides may exhibit more connections to receptors or proteins in order to trigger antitumor events [13]. For instance, the schizophyllans with MWs values ranging from 100 kDa to 10^4^ kDa exhibited a remarkable antitumor effect [40], and another immune-mediated antitumor polysaccharide SCG (polysaccharide from the mushroom *Sparassis crispa)* was found to have an MW value exceeding 2000 kDa [39]. It should be noted here that even though MW has been recognized as a critical characteristic to indicate the capacity to induce a reaction in immune systems [41], having higher MW is not necessarily always better than low MW in terms of anticancer activity [13].

In Figs. 5 and 6, the cell populations of HeLa and MCF-7 displayed significantly higher percentages for the sub-G1 stage compared to that of the control with PBS (*P* < 0.05). This indicates that the numbers of apoptotic cells were significantly higher in the treated samples [42]. Moreover, reduction in the population of treated cells in the G1/G0, S and G2/M phases compared to those of the controls suggests that a non-phase-specific cell cycle arrest effect of the tested polysaccharides [43]. In general, there has been only a limited understanding of the relationship between chemical properties of polysaccharides and their mechanisms in cytotoxicity toward cancer cells. The mechanisms of the anti-proliferative activities of polysaccharides were previously reported to include cell cycle arrest, induction of apoptosis, inhibition of motility, anti-angiogenesis, and anti-mutagenesis [1]. The results of a cell cycle analysis by PI flow-cytometry in the present study suggested that partially purified IPS and crude IPS would considerably induce apoptosis in HeLa and MCF-7 cells, respectively. It should be noted that the apoptosis effects of β-D-glucan from the fungus *Auricularia auricular-judae* have been reported [44]. In the latter study, the β-D-glucan was found to induce in vitro apoptosis on Acinar cell carcinoma and in vivo effect on Sarcoma 180 solid tumor cells. The apoptosis effect of this β-D-glucan was exhibited by up-regulation of Bax and down-regulation of Bcl-2 expression [44].

## Conclusions

This present study found that EPSs and IPSs isolated from microplasmodia of *P. polycephalum* had different chemical properties and cytotoxicity activities. Partially purified EPSs were composed mainly of galactose (80.22%), glucose (13.84%), mannose (5.70%), and rhamnose (0.24%), with an average MW of 14,762 kDa, while partially purified IPSs consisted mostly of glucose (86.46%), galactose (13.22%), mannose (0.23%), and rhamnose (0.09%), with an average MW of 1788 kDa.

The IPSs (both crude and partially purified) displayed significant toxicities against Hela cells, and samples of the partially purified IPS showed higher activity than the crude samples However, the purification process significantly reduced the cytotoxicity of the crude IPS toward MCF-7 as only 34.76% of MCF-7 cells remained viable after being treated with the crude IPSs, but almost no activity was recorded with the partially purified IPSs at the concentration of 1 mg/ml. The cytotoxicity of the crude IPSs 1 mg/ml toward MCF-7 was comparable with that of doxorubicin 50 μg/ml as the positive control. It is noteworthy that both IPS samples showed no negative effects toward the non-transformed cell line (BAEC). The crude EPSs seemed be the most effective agents against HepG2 cells, as they reduced the viability of these cells to 49.39%. However, almost the same level of cytotoxicity toward normal cells (BAEC) also was observed.

Cancer cell cycle analysis by PI flow-cytometry in the HeLa cell population treated with the partially purified IPS and the MCF-7 cell population treated with the crude IPS revealed that the IPS samples induced apoptosis the respective cancer cells.

## Methods

### Materials

The strain of *Physarum polycephalum* used for this study was purchased from Carolina Biological Supply Company (Burlington, North Carolina, USA).

Inoculum of *P. polycephalum* microplasmodia was prepared in an ATCC medium with an addition of a basal salts solution. One liter of the inoculum broth contained 100 ml of the basal salts solution, 10 g glucose, 10 g tryptone, 1.5 g yeast extract, and 900 ml of distilled water adjusted to pH 4.6. One liter of basal salts solution was composed of 40.4 g citric acid. H_2_O, 20.0 g K_2_HPO_4_, 6.0 g MgSO_4_.7H_2_O, 6.0 g CaCl_2_.2H_2_O, 0.6 g FeCl_2_.4H_2_O, 0.34 g ZnSO_4_.7H_2_O, 0.84 g MnCl_2_.4H_2_O [45] and 1000 ml distilled water.

The nutrient medium used for *P. polycephalum* microplasmodia growth and EPS and IPS production had a composition similar to the inoculum medium except that 20 g glucose, 6.59 g tryptone, and 3 g yeast extract were used instead [46].

Cancer cell lines (breast carcinoma MCF-7, liver carcinoma HepG2, cells and cervical carcinoma HeLa cells were purchased from the American Type Culture Collection, Manassas, Rockville, USA) and the non-transformed bovine aortic endothelial cells (BAECs, purchased from RIKEN, Tsukuba, Japan) were grown in DMEM 5% FBS medium at 37 °C in a 5% CO_2_ incubator.

### Microplasmodial culture of *P. polycephalum*

*Physarum polycephalum* was cultured in the nutrient medium broth as described by Truong et al. [46]. In brief, a volume of 10 ml of a 4-day old culture of *P. polycephalum* microplasmodia was transferred into a flask containing 100 ml of the nutrient medium broth. The culture was then incubated in the dark at 25 °C for 5 days.

### Collection of *P. polycephalum* microplasmodial crude EPS and IPS

For crude EPS collection, the 5-day-old microplasmodial culture was centrifuged and the microplasmodia pellet was separated from the supernatant. This pellet was later used for IPSs extraction. Chilled ethanol was then added into the supernatant (ratio 3:1, v/v) and the mixture was left overnight at 4 °C. The following day, the mixture was then centrifuged at 8500 rpm, 25 °C for 20 min. The pellet was collected and dried at 60 °C [14] and this dried pellet was referred to as the crude exopolysaccharide (crEPS).

For crude IPS (crIPS) extraction, the microplasmodial pellet collected as described above was added to sterile distilled water (ratio 1:3 v/v) and the mixture was then sonicated with a power of 240 W (20s on, 10s off) for 3 mins for complete cell disruption and to release the intracellular components. After sonication, the resulting cell lysate was centrifuged at 6000 rpm, 25 °C for 15 min and the supernatant was collected. Chilled ethanol with the same ratio was added for the IPS precipitation. The IPS sample was then collected and dried following the same procedure as described above.

### Partial purification of polysaccharides by deproteination and dialysis

To partially purify the samples, the crude IPS/EPS samples were dissolved in 5% (w/v) trichloroacetic acid (TCA) and left overnight at 4 °C for protein precipitation. The precipitated proteins were then discarded after centrifugation at 6000 rpm, 25 °C for 5 min. The supernatant containing either the EPS or IPS was sequentially dialyzed by subjecting to a 3.0 kDa molecular weight cut-off membrane against double distilled water three times to release all TCA and impurities until a neutral pH in the released water was recorded; then the dissolved polysaccharides were precipitated by adding chilled ethanol (ratio 3:1 v/v) and centrifuged at 8500 rpm, 25 °C for 20 min [47]. The pellet, referred to as partially purified intracellular polysaccharides (ppIPS) or partially purified exopolysaccharides (ppEPS), was dried at 60 °C and stored at 4 °C for bioactivity assessment.

### Total carbohydrate, protein, and sulfate group content analysis

The total carbohydrate content of the crude and partially purified EPS or IPS samples was measured by the phenol-sulfuric acid method using a D-glucose standard curve [19]. The protein and sulfate group contents were determined by the Bradford and barium chloride-gelatin methods, respectively [20, 21].

### GPC analysis

Average molecular weight (Mw) of the partially purified samples of EPS and IPS were determined by *gel permeation chromatography (GPC,* Agilent 1100 series system, Waters Ultrahydrogel 2000 column, Germany) with a refractive index (RI) detector. The samples were dissolved to the concentration of 1 mg/ml and then injected in the GPC instrument. The temperature of the column was held at 40 °C.

The mobile phase consisted of 5 mM Na_2_CO_3_ and 10 mM NaHCO_3_ at a flow rate of 1 ml/min. The GPC system was calibrated using a 380 kDa pullulan standard and duplicate injection of a sample was selected randomly (Supplementary Figure S2).

GPC data were created by using the add-on Rev.B.01.01 software (Agilent) with the standard curve prepared from pullulan standards with various molecular weights (23.7, 48, 100, and 380 kDa).

The polydispersity index (PI) was calculated from the Mw/Mn ratio, where Mn is number average molecular weight.

### ATR- FTIR spectroscopic analysis

Fourier transform infrared (FT-IR) spectroscopy was used for detecting major functional groups of each sample. Infrared spectra were recorded with Fourier transform infrared spectrometer (FTIR) Tensor 37 (Bruker) between 4000 and 600 cm ^− 1^. Samples were analyzed in powder form in the attenuated total reflection mode (ATR) with a diamond crystal. The number of scans was 64, and the resolution was 4 cm ^− 1^ [48].

### Monosaccharide composition analysis

The polysaccharide samples were first hydrolyzed by triflouroacetic acid (TFA), and then the obtained monosaccharides were derivatized with 1-phenyl-3-methyl-5-pyrazolone (PMP). Finally, PMP-derivatives of hydrolysate of samples were analyzed by LC-UV on an Agilent 1200 series according to the procedure described by Dai et al. [28].

### Evaluation of cytotoxicity activities of polysaccharides samples toward cancer and normal cell lines

The cytotoxicity activities of the crude and partially purified polysaccharides were evaluated using a MTT-based assay [49] against three cancer cell lines (MCF-7, HepG2, and HeLa) and a normal cell line (BAEC).

The cancer and non-transformed cells in 180 μl of DMEM medium were seeded into a 96-well microplate at a concentration of 5000 cells per well and cultivated under standard conditions for 24 h. A volume of 20 μl of serially diluted solution of the crude polysaccharides samples in phosphate buffered saline (PBS) was added into each well and incubated for another 24 h. Each well was then washed with 100 μl PBS. Then 50 μl of DMEM medium that contained 2 mg/ml MTT was transferred to each well. A volume of 200 μl of DMSO was then added to dissolve MTT formazan after 4 h and the light adsorption of the mixture was measured at 540 nm. PBS and Doxorubicin (50 μg/mL) were used as negative and positive controls, respectively.

Viability (%) was calculated according the following formula:

Viability (%) = [OD _sample_/ OD _control_] × 100%.

OD _sample_ and OD _control_ represent the absorbance of the test sample and the negative control, respectively.

### Cell cycle analysis by flow cytometry

Cell cycle analysis of the cell populations after being treated with the polysaccharide sample was carried out using the flow cytometry technique as described by Nguyen and Ho-Huynh [43]. In brief, concerned cancer cells were seeded into 3.5 cm Petri dishes at a density of 2 × 10^5^ cells/dish. After 24 h of growth, cells were treated with polysaccharide at the concentration of 1 mg/ml, then the dish was incubated for another 48 h. The cancer cells were then harvested, washed, and fixed with 70% ethanol for at least 2 h. The cell cycle profile was analyzed at 10,000 events by the BD Accuri C6 Plus flow cytometer (BD Biosciences). Propidium iodide (PI) 5 μg/ml was used for DNA labeling. The PI solution was prepared in PBS 1X contained Triton X-100 0.1% (v/v) and RNase 200 μg/ml.

The cell cycle profile of the tested cancer cells treated with PBS solvent was also recorded by the same protocol and used as a negative control in each test.

### Statistical analysis

The results of experiments were represented by their means ± SD (standard deviation). All the experiments were carried out in duplicate except for the GPC, LC-UV and FTIR tests. Statistical analysis was performed using a one-way ANOVA (SPSS 23). Differences were considered significant if *p* < 0.05.

## Supplementary Information

**Additional file 1.**

**Additional file 2: Supplementary Figure S2**: Overlay chromatogram of the partially purified EPS and IPS samples and 380 kDa pullulan as the control

## Data Availability

All data generated or analysed during this study are included in this published article and Supplementary Data S1 and Supplementary Figure S2 files. Supplementary Data S1 include data on cell cycle analysis by flow cytometry. Supplementary Figure S2 shows overlay chromatogram of the partially purified EPS and IPS samples and 380 kDa pullulan as the control.

## Abbreviations

ATR: Attenuated total reflection
DMEM: Dulbecco’s modified eagle medium
EPS: Exopolysaccharide
FTIR: Fourier-transform infrared spectroscopy
GPC: Gel permeation chromatography
IPS: Intracellular polysaccharide
LC: Liquid chromatography
MTT: 3-(4,5-Dimethylthiazol-2-Yl)-2,5-Diphenyltetrazolium Bromide
MW: Molecular weight
OD: Optical density
PBS: Phosphate buffered saline
PI: Propidium iodide
UV: Ultraviolet.
