# Supplementary material for: Cytotoxicity activities and chemical characteristics of exopolysaccharides and intracellular polysaccharides of Physarum polycephalum microplasmodia
Source: BMC Biotechnol. 2021 Mar 27;21:28. doi: 10.1186/s12896-021-00688-5 (PMC8005236; doi:10.1186/s12896-021-00688-5)
Supplement: Supplementary file 2 — Additional file 2: Supplementary Figure S2: Overlay chromatogram of the partially purified EPS and IPS samples and 380 kDa pullulan as the control [file 12896_2021_688_MOESM2_ESM.pdf]

Cytotoxicity activities and chemical characteristics of exopolysaccharides and intracellular polysaccharides of *Physarum polycephalum* microplasmodia

Tuyen TH Do[1,2,3], Tran NB Lai [1,2], Steven L Stephenson[4] and Hanh TM Tran [1,2 *]

***Corresponding author. E-mail: ttmhanh@hcmiu.edu.vn*

[1]School of Biotechnology, International University, Ho Chi Minh City, Vietnam

[2]Vietnam National University, Ho Chi Minh City, Vietnam

[3]Ho Chi Minh City University of Food Industry, Ho Chi Minh, Vietnam

[4]Department of Biological Sciences, University of Arkansas, Fayetteville, Arkansas

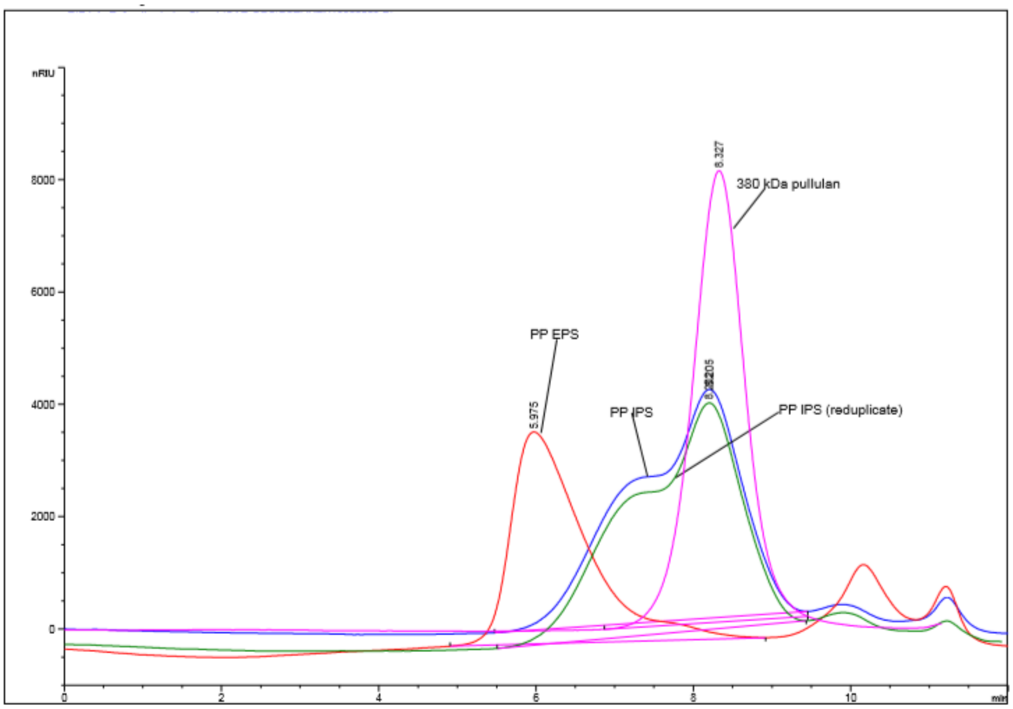

Supplementary Figure S2: Overlay chromatogram of the partially purified EPS and IPS samples and 380 kDa pullulan as the control
